# Origin, Purpose and Destiny of Man

**Published:** 1891-09

**Authors:** 


Origin, Purpose, and Destiny of Man; or, The Philosophy of the Three Ethers. By William Thornton. 12mo, pp. 100. Boston: Published by the author. 1891.

Some idea may be gathered of the purposes of the author of this book, if we quote a paragraph or two from his introduction. Thornton says :

> Life I call the first ether which is a continuous aggregate ; the second ether I call a composition of the potentialities heat, light, electricity and magnetism ; mechanical power being manifested during the activities of these potentialities. The third ether is a material nucleus which permits of the action of the other two ethers. All bodies manifesting the second and third ethers independently of the first, make up all inorganic bodies. Organized bodies require all three ethers. These two conditions constitute all things natural and supernatural.

In the first chapter is discussed the philosophy of the three ethers ; in the second, how to make medicine a science ; in the third, the germ theory of disease ; in the fourth, the transmission theory of disease ; in the fifth and last, immortality. It is difficult to pass such a book in critical review, without devoting more time and space than we have at command, but we can assure our readers of an interesting afternoon if they chose to occupy it in reading the volume. Whether it brings one nearer to the knowledge of the infinite, we leave to the judgment of each individual who communes with the author.

